# NFPscanner: a webtool for knowledge-based deciphering of biomedical networks

**DOI:** 10.1186/s12859-017-1673-1

**Published:** 2017-05-18

**Authors:** Wenjian Xu, Yang Cao, Ziwei Xie, Haochen He, Song He, Hao Hong, Xiaochen Bo, Fei Li

**Affiliations:** 1 Department of Biotechnology, Beijing Institute of Radiation Medicine, 27 Taiping Street, Haidian District, Beijing, 100850 China; 2 Tianjin Institute of Health & Environmental Medicine, 1 Dali Road, Heping District, Tianjin, 300050 China; 3 Department of Biomedical Engineering, College of Life Science and Technology, Huazhong University of Science and Technology, 1037 Luoyu Road, Wuhan, 430074 Hubei China; 4 Department of Biomedical Engineering, National University of Defense Technology, 109 Deya Road, Kaifu District, Changsha, 410073 Hunan China

**Keywords:** Network deciphering, Pathway analysis, Online interactive analysis, Network fingerprint

## Abstract

**Background:**

Many biological pathways have been created to represent different types of knowledge, such as genetic interactions, metabolic reactions, and gene-regulating and physical-binding relationships. Biologists are using a wide range of omics data to elaborately construct various context-specific differential molecular networks. However, they cannot easily gain insight into unfamiliar gene networks with the tools that are currently available for pathways resource and network analysis. They would benefit from the development of a standardized tool to compare functions of multiple biological networks quantitatively and promptly.

**Results:**

To address this challenge, we developed NFPscanner, a web server for deciphering gene networks with pathway associations. Adapted from a recently reported knowledge-based framework called network fingerprint, NFPscanner integrates the annotated pathways of 7 databases, 4 algorithms, and 2 graphical visualization modules into a webtool. It implements 3 types of network analysis:

- Fingerprint: Deciphering gene networks and highlighting inherent pathway modules
- Alignment: Discovering functional associations by finding optimized node mapping between 2 gene networks
- Enrichment: Calculating and visualizing gene ontology (GO) and pathway enrichment for genes in networks

Users can upload gene networks to NFPscanner through the web interface and then interactively explore the networks’ functions.

**Conclusions:**

NFPscanner is open-source software for non-commercial use, freely accessible at http://biotech.bmi.ac.cn/nfs.

**Electronic supplementary material:**

The online version of this article (doi:10.1186/s12859-017-1673-1) contains supplementary material, which is available to authorized users.

## Background

Researchers have widely used high-throughput technologies such as microarrays, next-generation sequencing and proteomics to generate differential expression profiles. In the context of gene networks, biological network analysis tools and web servers can identify, infer, reconstruct, and visualize these changes, which helps biomedical scientists generate context-specific molecular networks [1–7]. Such networks have both gene nodes and gene interaction edges. Further annotating the molecular networks with existing knowledge helps better explain the experimental findings. Many tools focus on enrichment analysis of networks’ node lists in terms of gene ontology (GO) annotations, pathway genes or disease signature gene membership; however they cannot take network edges and interaction events of classic signaling pathways into account [6, 8–13]. No currently available tools can explore the functions of molecular networks without losing this edge information, even though edges and nodes can be successfully managed via global network alignment algorithms to facilitate knowledge transfer across species [14–17].

Because pathways curated by domain experts essentially describe context-specific gene interactions in certain biological processes, we have redefined these “pathways” as gene network modules. Thus, a network is viewed as an organization of multiple “pathway” network modules. Resources of well-annotated pathways serve as the gold-standard reference for basic network modules; any other networks can be annotated in the coordinates of these reference networks.

In the “network fingerprint” framework, a biomedical network (or “query network”) is characterized as a spectrum of numerical representations by making systematic comparisons with reference networks [18]. The essence of network fingerprint extraction is generating similarity scores between a query network and each reference network by a 3-step procedure: “network merging,” “node clustering,” and “similarity scoring.” The result of the first 2 steps is node-node mapping between 2 networks, which is quite similar to the output of network alignment algorithms [19]. Network alignment can easily be adapted into network fingerprinting, and the original 3-step procedure condensed to 2 steps: “node-node mapping” and “similarity scoring”. Although existing network visualization and alignment tools provide alignment details for 2 networks using various node mapping criteria (common GO terms, coding gene sequence similarities, and protein sequence similarities), none of them quantifies similarities in large-scale analysis [14, 16, 20–26].

Thus, we introduced a webtool for network fingerprint analysis, NFPscanner (Network FingerPrint scanner). This tool implements 4 node-node mapping algorithms (IsoRankN [16], SPINAL [17], GHOST [14], and APCluster-based method [27]), 2 similarity scoring metrics, reference network sets from 7 pathway databases (KEGG [28], Reactome [29], NCI [30] etc.), and 2 visualization modules. NFPscanner is advantageous in several specific ways:

- It implements more network alignment algorithms than the original network fingerprint framework.
- It extends the sources of reference networks.
- It provides a user-friendly interface and a one-stop network deciphering solution.

NFPscanner is compatible with gene lists from common differential expression analysis, as many popular web servers can expand a gene list into an “NFPscanner-acceptable” gene network format [1, 5, 7].

## Implementation

**Fig. 1:**
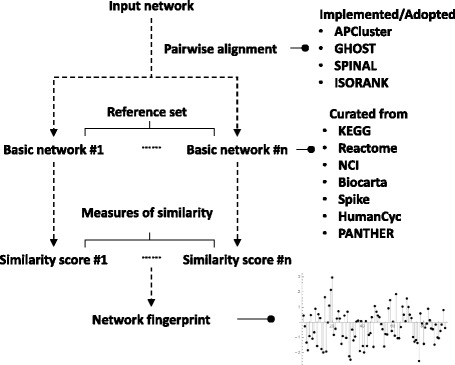
Typical NFPscanner workflow. Network fingerprint analysis of an input network consists of a series of pairwise alignments with basic networks. The network fingerprint is represented in a vector of similarity measures between the input network and different basic networks

NFPscanner is designed to decipher the potential functions of query networks on the basis of reference networks representing different biological processes. Users can upload up to 5 query networks as input data, specify a reference network set, start the analysis to extract network fingerprints, monitor the computation progress of background tasks, and visualize the fingerprint outputs when analysis is complete (Fig. 1). For demonstration purposes, interactive step-by-step tutorials on an example analysis are provided on the “Network Fingerprint Scan” (http://biotech.bmi.ac.cn/nfs/networksimilarityAnalysis?type=1) and “Pairwise Alignment” (http://biotech.bmi.ac.cn/nfs/networksimilarityAnalysis?type=2) webpages. The NFPscanner website supports popular browsers such as Internet Explorer v.11, Chrome v.54, Firefox v.43, Safari v.5 and Opera v.40.

### User’s guide on input data preparation and parameter settings

NFPscanner supports analysis of human gene networks, whose ID can be Entrez Gene [31], UniProt [32], Gene Symbol, Ensemble gene ID [33], RefSeq ID [34] and KEGG ID. Acceptable input network formats include edge list, which is a list of network edges, and GraphML, which is an XML-based file format for networks [35]. Users can convert other network formats to edge list using the igraph tool [36]. Gene lists from differential expression analysis should be expanded as gene networks in the context of landscape interactomes, as described on the “Help” page (http://biotech.bmi.ac.cn/nfs/networksimilarityHelp). The recommended network size is 50–100 nodes and 100–1,000 edges.

Once input data have been uploaded successfully, NFPscanner shows a preview of query networks as confirmation. Users then set program parameters, including similarity measures (default: GO), permutation cycles (default: 10 randomized networks generated for computing normalized Z-scores), node-mapping algorithms (default: APCluster), and reference networks from a resource list of predefined basic networks. They can adjust algorithm-specific parameters in the “Advance Parameters” panel if they desire. Finally, they click the “Submit” button to start analysis. They can bookmark the “Results” page or opt to receive notification emails to monitor running status. To provide an example of job execution time, if example Network #2 “upregulated gene networks in neonatal sepsis” (provided in the “Network Fingerprint Scan” module) is analyzed with the default “APCluster” algorithm and parameters, analysis is completed in 0.12 h in fast mode and 1.33 h in normal mode.

### User’s guide on interpretation of analysis results

Once analysis is complete, NFPscanner redirects users to an interactive webpage providing the results of the network fingerprint. There are 3 types of interactive analysis modules:

1. **Fingerprint scan** deciphers gene networks and highlights inherent pathway modules. The results page shows 2 panels side-by-side: “Fingerprint Graph” and “Fingerprint Data.” Fingerprints corresponding to multiple input networks are visualized in a multicolor graph whose vertical axis indicates similarity scores and whose horizontal axis indicates categories of reference networks. Mouseover on a data point shows the corresponding similarity scores between the input network and some reference networks. If multiple fingerprint curves differ greatly in certain reference networks, the input networks have different functional associations with those biological pathways.
2. **Pairwise alignment** discovers functional associations by finding optimized node mapping between 2 gene networks. After fingerprint curve analysis, users may identify some data points of high similarity scores. They can click the corresponding data point to open the pairwise alignment module below the fingerprint panel.
  The new “Alignment View” displays nodes in color, based on clusters of information output from node mapping algorithms. Gene nodes are hyperlinked to ID entries in external databases, including NCBI Gene [31], GeneCards [37], UniProt, HGNC genenames [38], Ensembl, NCBI RefSeq. In the right-hand multi-tab panel “Alignment & Enrichment Data,” the “Network Alignment” tab lists similarity scores of gene clusters. Select a gene cluster, and the corresponding genes will be highlighted in the left-hand “Alignment View” panel.
  Pairwise alignment analysis is a useful tool for discovering representative gene clusters in 2 networks. It is also available as an independent module on the “Pairwise Alignment” webpage (http://biotech.bmi.ac.cn/nfs/networksimilarityAnalysis?type=2). Users can upload 2 input networks to the webpage and run the analysis to generate an alignment view without a network fingerprint.
3. **Enrichment analysis** calculates and visualizes GO and pathway enrichment for gene sets in networks. Besides the previously mentioned “Network Alignment” tab, the “Alignment & Enrichment Data” panel has 2 more tabs: “Pathways,” which lists the enriched KEGG pathways with genes from 2 networks, and “Gene Ontology,” which lists the enriched GO terms with genes from those same 2 networks. The “Alignment & Enrichment Data” panel is a custom visualization tool coupled with “Pairwise Alignment” analysis. It categorizes gene sets by enriched GO terms or enriched pathways and visualizes enrichment results in “Alignment View” with genes colored by different GO terms. When the user selects the “pathway” of interest from the enrichment results table, NFPscanner highlights that pathway’s genes.

These 3 types of analysis can be seamlessly used in a workflow to help investigate gene networks. Users can follow fingerprint analysis with a series of pairwise alignment analyses, and each alignment analysis can help visualize enrichment analysis of genes. Users can also sort and search on all of the above types of interactive analysis modules by keywords to find desired pathways and GO terms (Fig. 3).

Finally, although the output of the network fingerprint is described as an interactive webpage, it can be exported as images and PDFs via the downloading icons in each panel. Additional information about user interface, parameter settings and other features of NFPscanner are listed in Additional file 1.

## Methods

### Network fingerprint framework

The generalized network fingerprint framework has 2 steps, “node-node mapping” and “similarity scoring.” The framework compares a query network with a series of reference networks, then quantifies the similarity scores between the query and each reference network. It outputs an array of similarity scores that represent the query network as unique spectrum called “network fingerprint.” The network fingerprint of a query network indicates its relative similarities with the reference networks.

More importantly, the similarity between 2 networks is represented as a normalized Z-score inferred from gene interactions and GO terms or sequences of the networks’ genes. If 1 query network is being compared with a set of reference networks, those reference networks with high similarity scores are believed to have close biologically relevant connections with the query network. The network fingerprint lets the user annotate the query network. If multiple query networks are being input, each of them can produce a unique spectrum that provides standardized pathway-level evidence for differences between those query networks. Furthermore, if the fingerprints of multiple query networks are each computed with the same set of reference networks, the fingerprint patterns can help classify those query networks and explain their mechanisms. For example, if the user extracts fingerprints of several types of abnormal gene networks associated with several types of cancer in reference to KEGG signaling pathways, they can compare patterns of abnormal signaling events in all pathways. Therefore, network fingerprinting is an intuitive solution to discovering network characteristics at the pathway level.

### Node mapping using affinity propagation clustering and network alignment approach

In addition to affinity propagation clustering described [18] as the prototype approach, we also implemented a new approach based on network alignment algorithms to find optimized node-node mapping between two networks. The node mapping information eventually affects the computation of corresponding similarity scores between 2 networks. NFPscanner uses the APCluster-based method as the default algorithm, and also allows users to choose 1 alternative alignment algorithm: IsoRankN, SPINAL, or GHOST.

### Similarity scoring system

Another feature of network fingerprint analysis is the similarity scoring system, which plays roles in both the “node mapping” and “similarity scoring” steps. The similarity scoring system determines whether the user assigns the edge weight of 2 networks based on semantic similarity between GO terms or gene sequence similarity between interaction genes. In the node mapping step, the interaction genes with larger similarity scores tend to be mapped into a cluster. In the similarity scoring step, network similarity is the mean of all clusters’ local similarity scores, which are obtained using similarity scores of cluster genes as previously described [18]. Each analysis lets the user specify 1 preferred similarity score system in the “Parameter Setting” panel.

### Reference set of networks

We retrieved reference networks from 7 pathway databases: KEGG, Reactome, NCI, Biocarta [39], Spike [40], HumanCyc [41] and PANTHER [42] (Table 1). KEGG signaling networks were directly retrieved from a KEGG database using the R/Bioconductor package *KEGGgraph* [43]. The R/Bioconductor package *graphite* [44] provided networks from 6 other pathway databases: Reactome, NCI, Biocarta, Spike, HumanCyc and PANTHER. Pathways with fewer than 10 edges were excluded. Domain experts manually categorized the remaining 766 reference networks into 49 sets of NFPscanner reference networks (see details in Additional file 2). This predefined set of reference networks helps users conduct network fingerprint analysis on special research topics, such as regulatory circuits, signaling pathways, hormone regulation, diseases, and development. The reference sets listed on the “Resources” webpage are automatically ranked by historical usage count (http://biotech.bmi.ac.cn/nfs/networksimilaritystatistical).

**Table 1:** Organization of the current reference set of pathways in NFPscanner. We compiled 766 entries from 7 pathway databases — KEGG, Reactome, NCI, Biocarta, Spike, HumanCyc and PANTHER — into 49 biomedically relevant categories of basic networks, which are listed in the NFPscanner reference sets

	Source database							
Category	KEGG	Reactome	NCI/PID	SPIKE	HumanCyc	PANTHER	BioCarta	Total
Genetic Information Processing	7	22	40			5	19	93
Environmental Information Processing	27	6				16	29	78
Cellular Processes	13	23	9			25	46	116
Organismal Systems	61	14				26	28	129
Metabolism		12						12
Signal Transduction		23			1		83	107
Transport Processes		8						8
Biosynthesis					20			20
Degradation					10			10
Modification					15			15
Brain			11					11
Cell Adhesion			31					31
Cytokine and Chemokine			25					25
Development			7					7
Growth Factor			22					22
Hormone			16					16
Immmune Response			11					11
Kinase and Phosphatase			12					12
Ras Superfamily			11					11
Second Messengers			8					8
Transcription Factor			27					27
Cell Cycle				9				9
DNA Damage Response				8				8
Hearing Related Pathways				5				5
Programmed Cell Death				6				6
Total	108	108	199	28	46	72	205	766

### Design of web server

NFPscanner is implemented in Java and R scripts. The front-end of the web server is implemented in a Java Spring framework. Network visualization and interactive exploration modules are based on several open-source projects: Cytoscape web [45], Bootstrap, jsTree, D3.js, ECharts, and jsPDF. The back-end scripts are written in R language (v.3.2.2). GO enrichment was done with R package *clusterProfiler* [46]. For developer convenience and future upgradability, NFPscanner has a flexible built-in interface that permits users to plug in new algorithms and to add reference databases and other similarity scoring systems. We conducted a regular code review and a software test to validate the analysis system. The source codes for the web server are available at https://github.com/xuwenjian85/NFPscanner-webserver.

### Performance and validation of web server

Execution time of network fingerprint analysis varies with choice of algorithm and parameter settings. To compare the performances of different combinations of algorithms and parameters, we selected the upregulated subnetwork of neonatal sepsis [47] as standard input data, used “108 KEGG signaling pathways” as a standard reference set, and set up a series of experimental analyses. Additional file 3: Table S1 summarizes the execution times of these experiments, suggesting that the “APCluster” algorithm is most efficient, that 2 similarity measures (GO terms and gene sequences) have comparable performances, and that execution times are proportional to permutation cycles.

We selected KOBAS (v2.0) [8], the most common software for KEGG pathway enrichment analysis, to validate NFPscanner’s accuracy. To compare NFPscanner and KOBAS results, we used KEGG disease datasets as standard input datasets and KEGG signaling pathways as reference networks, ran network fingerprint analysis with NFPscanner, and ran KEGG pathway enrichment analysis with KOBAS. For each input disease network, we formulated the accuracy evaluation problem as binary classification, setting the labels of reference networks (signaling pathways) according to the KOBAS enrichment result (positive if corrected *p*-value < 0.05 and negative otherwise) and considering the network fingerprint scores as the prediction scores of these pathways. We then generated receiver operating characteristic (ROC) curve and area-under-the-curve (AUC) values for each input disease data using R package *ROCR* [48] (see details in Additional file 3: Table S2 and Figure S1), and found that the AUC of network fingerprint systems relative to pathway enrichment were 0.879 on average.

## Results

**Fig. 2:**
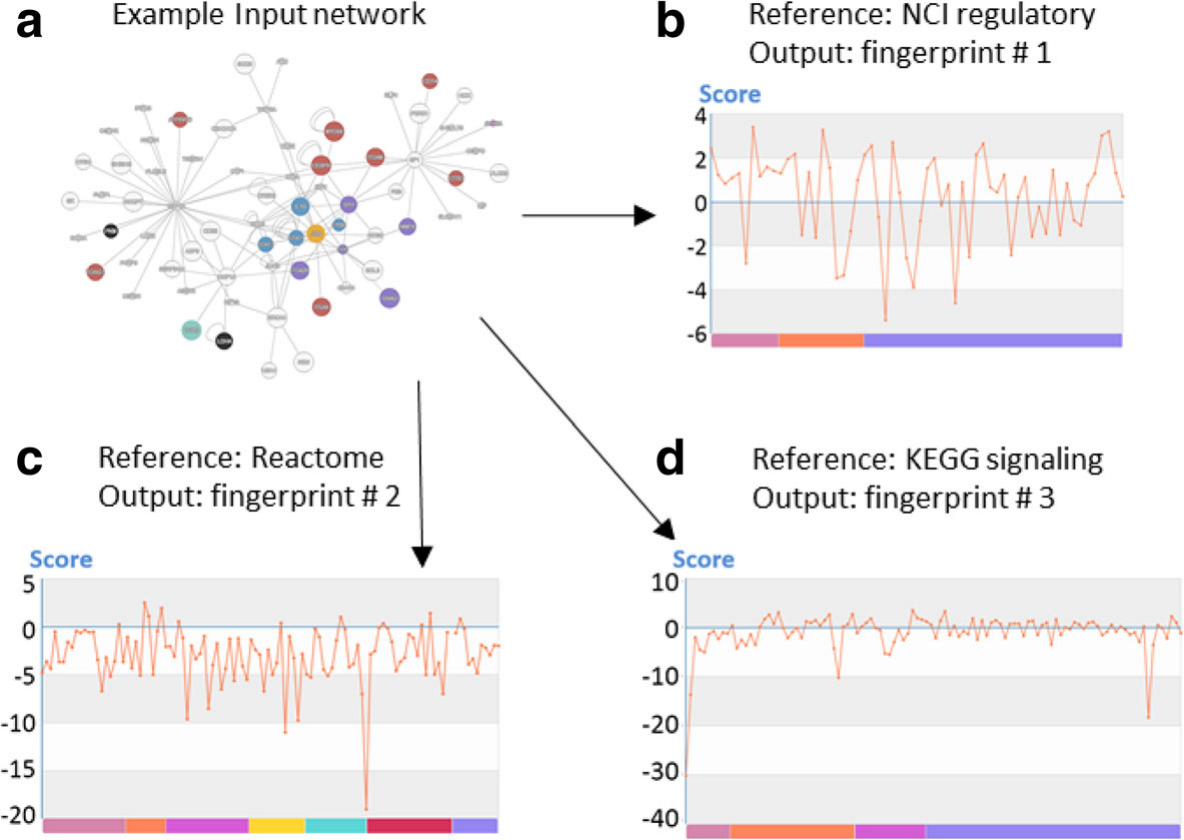
Example of NFPscanner fingerprint output, with a (**a**) neonatal sepsis upregulated subnetwork as input. We performed network fingerprint analysis of this disease-specific condition with 3 different reference sets of basic networks, **b** NCI regulatory pathways, **c** Reactome pathways, **d** KEGG signaling pathways, deciphering the input network from different biological perspectives. Each plot represents a spectrum-like vector of similarity measures between the input network and a set of basic networks

We applied NFPscanner as a downstream tool of the microarray analysis pipeline. In Smith et al.’s study [47], blood RNA profiling of 1 virus-infected patient, 27 sepsis patients, and 35 match controls was performed using the Illumina HT-12 platform. After several steps that involved statistical testing and filtering, they revealed 52 differential expression genes with stringent cutoffs (*adj.p* ≤ 10^−5^, fold change ≥ 4). With networks of differential genes derived from InnateDB [49], as well as by using the Cytoscape plugin jActiveModules [3], they identified a top-scoring upregulated subnetwork of neonatal sepsis (Fig. 2a). The network of 70 genes and 125 interactions was implicated by a systemic, unbalanced homeostatic immune response that underlay clinical signs. Next, we uploaded this network as input data and analyzed it in the “Network Fingerprint Scan” module under the parameter setting “similarity measure (Gene Ontology), permutation cycles (100), alignment algorithm (APCluster), and reference set (NCI regulatory networks).” Fig. 2b represents the output fingerprint as an interactive chart. The neonatal sepsis network was highly associated with immune pathways such as TLR signaling, TNF signaling, leukocyte transendothelial migration, FoxO signaling, and phagosome and platelet activation; this is in accord with the literature. Moreover, the fingerprint revealed other associated pathways, such as HIF-1, estrogen and prolactin signaling.

**Fig. 3:**
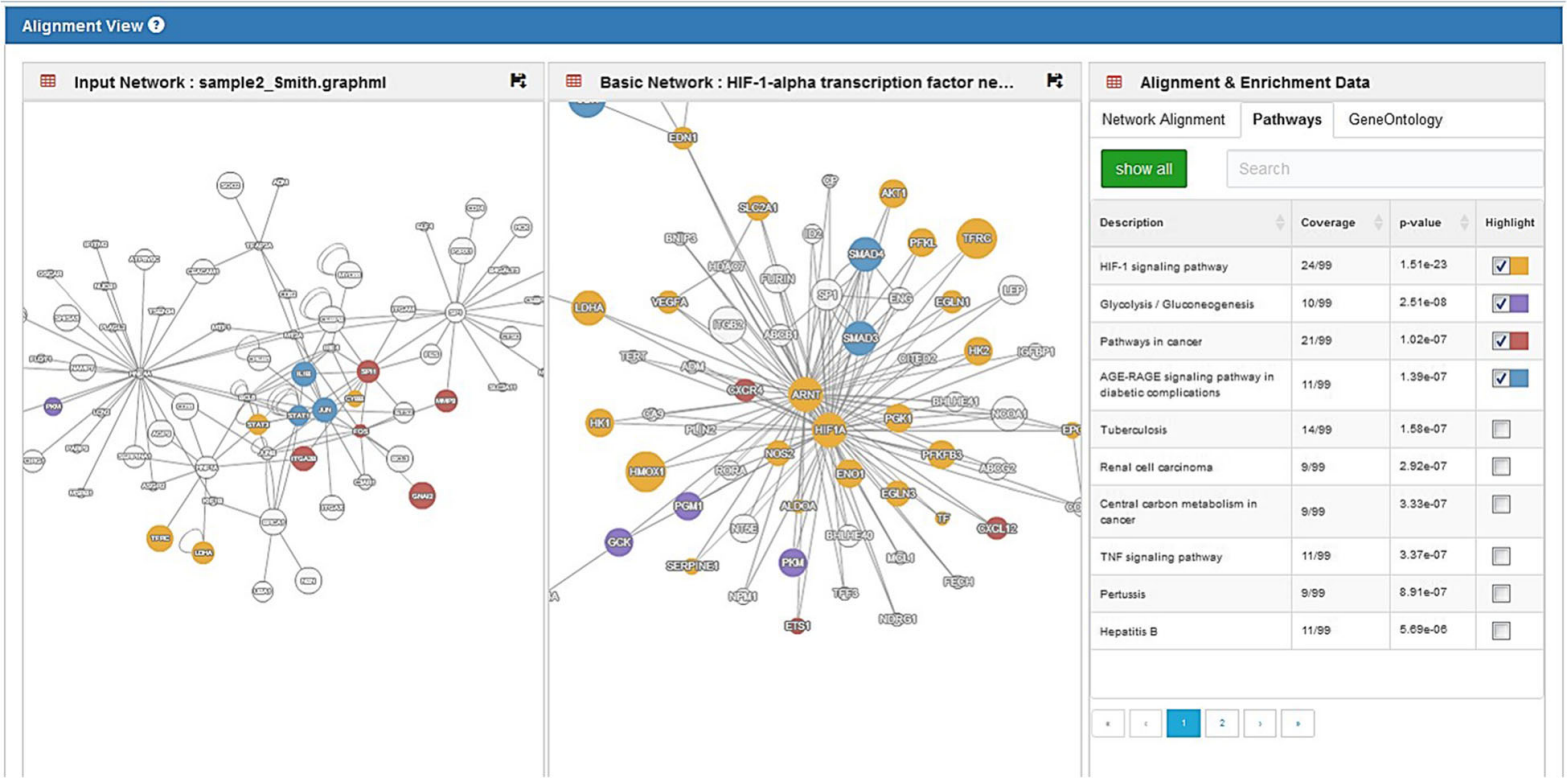
Example of a pairwise alignment view of a neonatal upregulated subnetwork and HIF-1-alpha transcription factor network. Nodes of both networks in most enriched pathways are shown in yellow (HIF-1 pathway), purple (glycolysis pathway), brown (cancer pathway), etc

If a user wants to know how the HIF-1-alpha transcription factor network participates in neonatal sepsis, they can click on the data point labeled “HIF-signaling” in the fingerprint view of the neonatal sepsis upregulated subnetwork. This expands the pairwise alignment view at the bottom of the webpage in default color settings. Switching to the “Pathways” tab on the right opens a list of enriched pathways. Since the user is interested in the “HIF-1 signaling pathway,” they would click on this entry to highlight the genes involved in HIF-1 signaling in both networks (Fig. 3).

We also applied our tool on manually curated metabolic network of obesity from Jagannadham’s study [50]. The network of 346 genes and 465 edges was uploaded as input data and analyzed by “network fingerprint scan” module under the parameter setting “similarity measure (Gene Ontology), permutation cycles (100), alignment algorithm (APCluster) and reference set (108 KEGG signaling networks)”. The network fingerprint result suggests that this obesity-related gene network is highly associated with cGMP-PKG signaling, Regulation of lipolysis in adipocytes, AMPK signaling, cAMP signaling, Adipocytokine signaling, Insulin signaling, PPAR signaling pathway, etc. Next, we analyzed the neonatal sepsis network with this parameter setting and we found it is highly associated with TLR signaling, Osteoclast differentiation, B cell receptor signaling, NF-kappa B signaling, HIF-1 signaling, and RIG-I-like receptor signaling. Lastly, we compared two network fingerprints side-by-side (see Additional file 3: Table S3), we concluded that obesity network and neonatal sepsis network have significantly different association with the above pathways except HIF-1 signaling.

## Discussion

NFPscanner serves as a bridge between various molecular networks and annotated functional pathways or modules. We believe it could attract wide interest from biomedical researchers, and plan to extend the reference sets to cover tissue-specific and species-specific topics in the future.

However, there are still a few limitations. Due to server capacity, a set of query networks must be uploaded separately rather than a whole dataset in “Network Fingerprint Scan” job. Furthermore, the analysis pipeline cannot accept user-defined reference sets. It would be beneficial to allow users to upload reference networks together with query networks and perform customized network fingerprint analysis. For now, users can contribute their customized networks as new entries in the public reference database by contacting the author team.

## Conclusions

NFPscanner provides a ready-to-use pathway-based network analysis resource with an intuitive user interface. It makes use of pathway knowledge and existing algorithms to compare multiple networks in the pathway coordinates in a novel and straightforward way.

## Availability and requirements

Project name: NFPscanner

Project home page: http://biotech.bmi.ac.cn/nfs

Operating system(s): Platform independent

Programming language: R and Java

Other requirements: Adobe Flash Player browser plugin

License: Creative Commons Attribution-NonCommercial 4.0 International License

Any restrictions to use by non-academics: NFPscanner is freely accessible for non-commercial users.

## Additional files

Additional file 1: Additional information about user interface, parameter settings and other features of NFPscanner. (DOCX 1228 kb)

Additional file 2: Additional information about 49 sets of reference pathways in the NFPscanner resource list, including pathway names, edge counts, and node counts. (ZIP 44 kb)

Additional file 3: Table S1. Performance of algorithm and parameter combinations on the same input data set. **Table S2.** AUC value of networks fingerprint results for 73 KEGG diseases datasets. **Figure S1.** Average ROC curves derived from **Table S2. Table S3.** Fingerprints data file in Case Studies. (ZIP 95 kb)

## Abbreviations

AMPK: 5’ adenosine monophosphate-activated protein kinase
APCluster: Affinity propagation clustering
FoxO: Forkhead box O
HIF-1: Hypoxia-inducible factor -1
HumanCyc: Encyclopedia of human genes and metabolism
KEGG: Kyoto encyclopedia of genes and genomes
NCI: National cancer institute
PANTHER: Protein analysis through evolutionary relationships
PKG: Protein kinase G
PPAR: Peroxisome proliferator-activated receptor
RIG-I: Retinoic acid-inducible gene I-like
SPIKE: Signaling pathway integrated knowledge engine
TLR: Toll-like receptor
TNF: Tumor necrosis factor
