# Supplementary material for: NFPscanner: a webtool for knowledge-based deciphering of biomedical networks
Source: BMC Bioinformatics. 2017 May 18;18:262. doi: 10.1186/s12859-017-1673-1 (PMC5437514; doi:10.1186/s12859-017-1673-1)
Supplement: Supplementary file 1 — Additional information about user interface, parameter settings and other features of NFPscanner. (DOCX 1228 kb) [file 12859_2017_1673_MOESM1_ESM.docx]

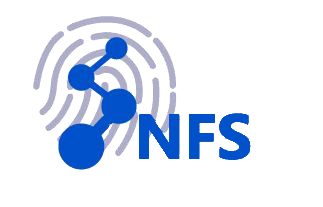


**Network Fingerprint Scanner**

**NFPscanner：a webtool for knowledge-based deciphering of biomedical networks**

#### Upload input networks

This section describes how to upload input networks to NFPscanner server. The procedure of uploading input networks is the same in "Network Fingerprint Scan" and "Pairwise Alignment" analysis. Here the former one is used as an example.
Open the NFPscanner "Home" page, and navigate to "Network Fingerprint Scan" tab. The NFPscanner analysis can extract fingerprint from single or multiple input networks. The format of input networks can be either edge list or graphml format. (Note: If you have gene lists instead of ready-to-use network files, please firstly read the previous two sections "Make an input network from a gene list" and "Make an input network from other network formats" and prepare the network files accordingly.)
Network data adapted from recent publications are provided as examples. You can try NFPscanner with examples.
In the "Input Networks" section, click "upload one network" button, click "Add file" to select input file from local disk, specify ID type, then click "OK" button. Otherwise, enter a network in textbox directly.


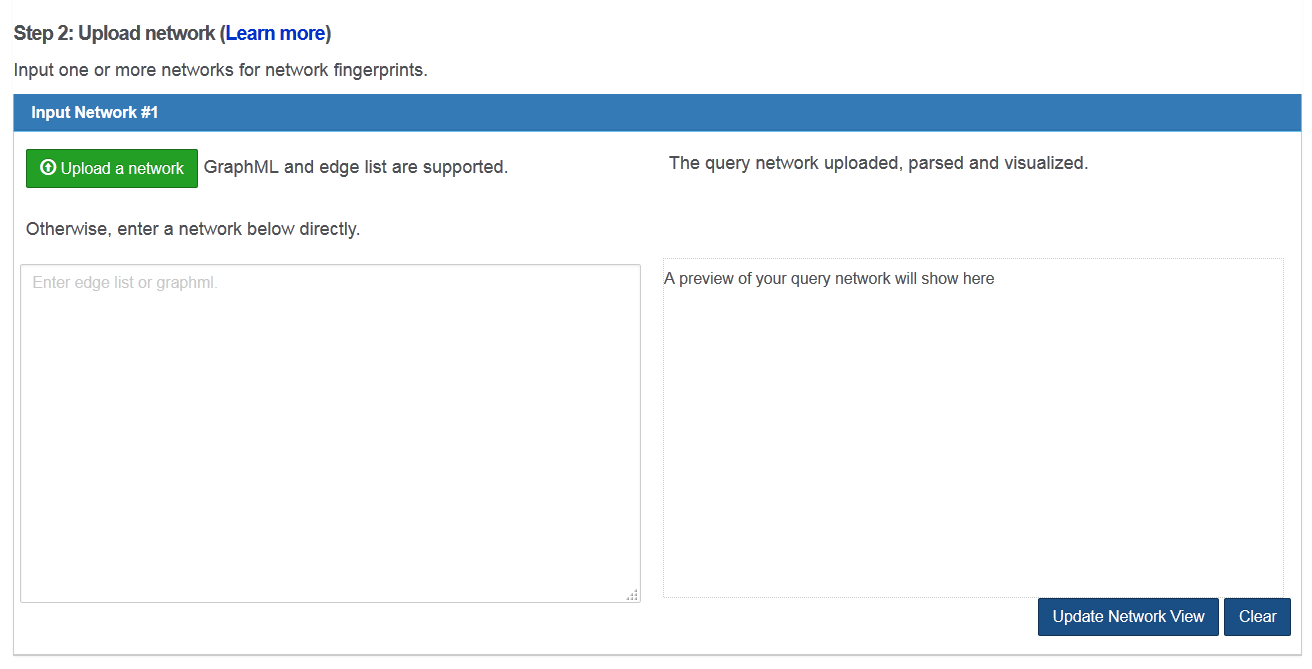


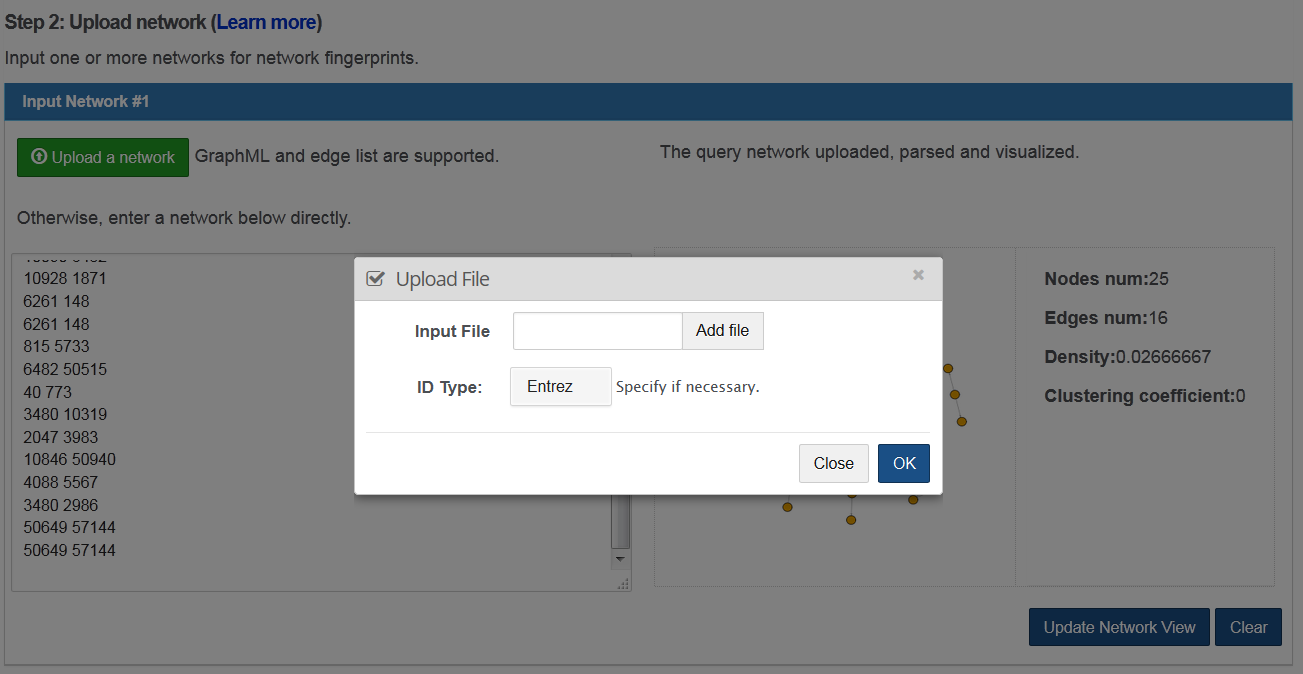


Click "Update Network View" button, verify format type and the ID type from dropdown list, then click "OK" button. A network graph is previewed. Now you have successfully uploaded one input network to NFPscanner.


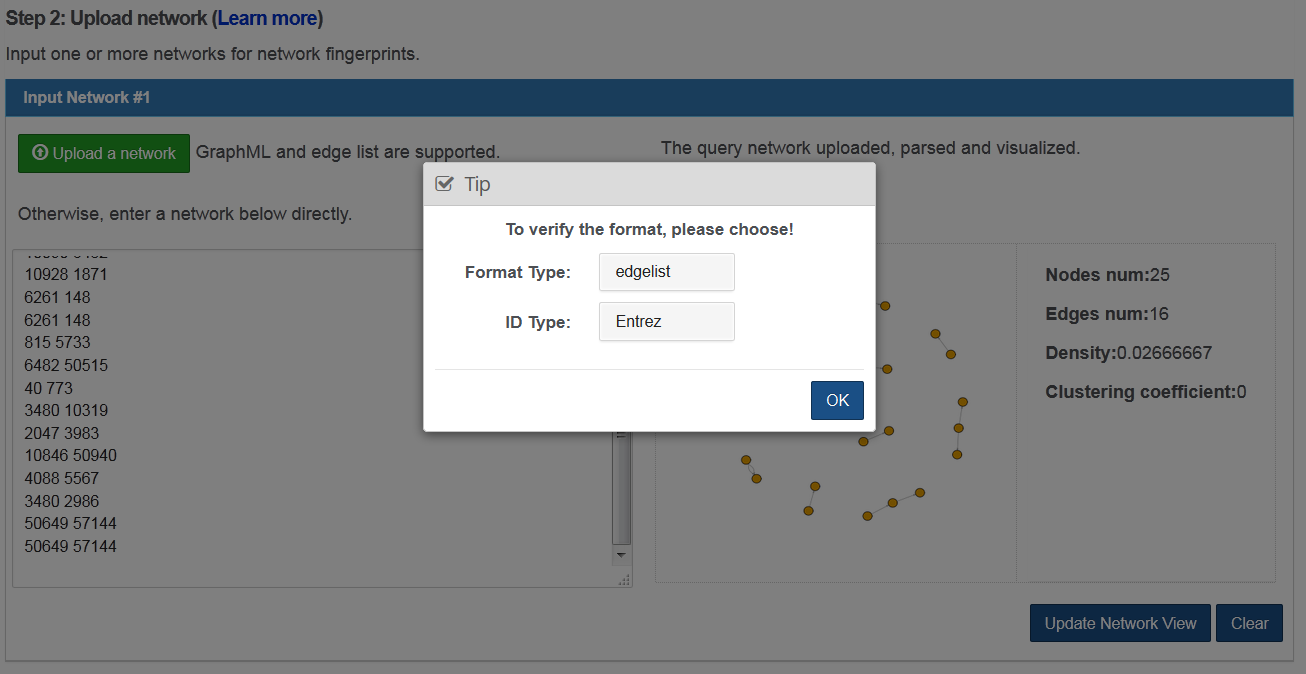


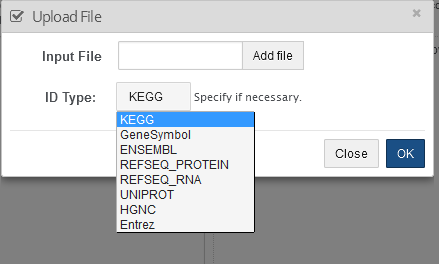


Optionally, if you want to scan network fingerprints of more than one networks, just click the "Add another network" button to open more input sections below. Up to five input networks per run are supported by NFPscanner web server.

#### Set parameters

This section describes how to set parameters for "Network Fingerprint Scan" analysis and "Pairwise Alignment" analysis. The general parameters is similar in both type of analysis. Here the former one is described as an example.
After input networks are uploaded and previewed successfully, scroll down the webpage to "General Parameters" section. To help users select parameters more productively, the meaning of each parameters is explained here.


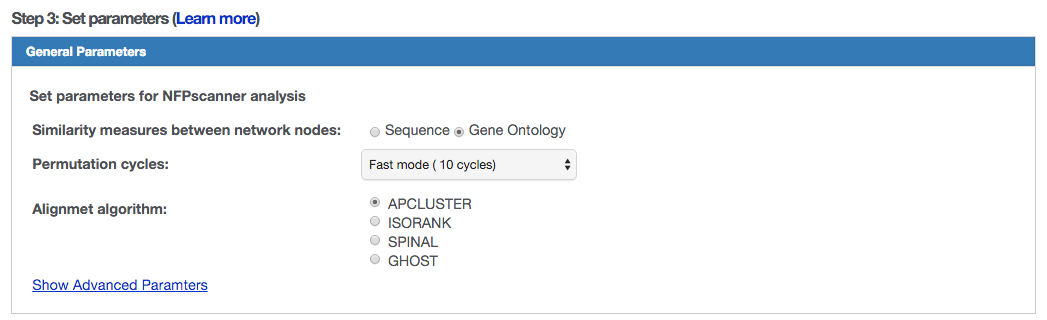


##### 1) Similarity measures between gene networks

Default setting of similarity measure is "Gene Ontology", which is based on sematic similarity of GO terms of genes. Alternatively, you can also select "Sequence" to use sequence blast scores as similarity measure. Both options are biologically meaningful. However, the network fingerprint of the same input network will be different if different similarity measure is chosen.

##### 2) Permutation cycles

The permutation of basic networks is the necessary step to calculate standardized similarity scores, which rule out the impact of size of reference basic networks. For a basic network, a sample size of 100 randomized networks is recommended to estimate the distribution of similarity scores and standardize similarity scores. Given an input network of 48 genes, the calculation time of 100 permutation cycles on one basic network takes ten minutes on NFPscanner web server. It is equivalent to say that NFPscanner may run 12 ~ 16 hours to return one network fingerprint if KEGG signaling pathways (contain 108 basic networks). However, 10 permutation cycles will reduce to a couple of hours for the above job. The estimated running time is summarized in following table. We encourage for all users to run initial run with the default setting "Fast mode (10 cycles)". After you evaluate the initial result, run 100 or 1000 permutation cycles when necessary.

**Estimated running time in different permutation cycles**

| Permutation cycles | Mode | Estimated running time (1 input network against reference set of 108 basic networks ) |
| --- | --- | --- |
| 10 | Fast | < 2 hours |
| 100 | Normal | 12 ~ 16 hours |
| 1000 | Accurate | Several days |

##### 3) Alignment algorithm

The four alignment algorithms are implemented on NFPscanner. These algorithms have similar functions and any of them can work as NFPscanner core alignment algorithm. Users can choose one from the list, and the default setting is "APCLUSTER". To learn more about each algorithms, please refer to the original publications of algorithms at [Citation](http://biotech.bmi.ac.cn/nfs/networksimilarityHelp#citationbodyA) section.

##### 4) Advanced parameters (Optional)

NFPscanner allow users to fine tune alignment algorithms through "Advanced parameters" panel. Theoretically, optimization of alignment algorithm performance is possible by adjusting advanced parameters for each algorithm. However, we recommended most users to keep the advanced parameters default.
To set advanced parameters, click on the "Show Advanced Parameters" button, and open the "advanced parameters" panel. Depending on the selected alignment algorithm in "General parameters" panel, the corresponding advanced parameters are shown in the new "Advanced parameters" panel.


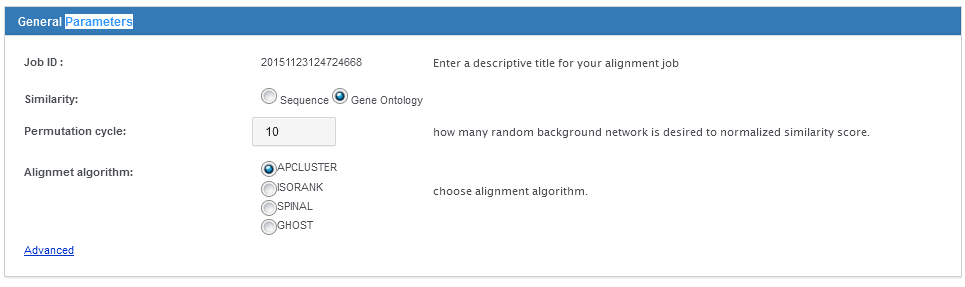


We list the short explanations for all provided parameters for users’ quick referenece. These information is based on adapted and modified from original program README files.

###### Advanced parameters for "APCLUSTER"

APCLUSTER of NFPscanner relies on R package "apcluster", which is an R implementation of Affinity Propagation clustering algorithm. It clusters data using a set of real-valued pairwise data point similarities as input. Each cluster is represented by a cluster center data point (the so-called exemplar). The method is iterative and searches for clusters maximizing an objective function called net similarity. Refer to [Citation](http://biotech.bmi.ac.cn/nfs/networksimilarityHelp#citationbodyA)for more details.

| q | If q=NA, exemplar preferences are set to the median of non-Inf values in s. If q is a value between 0 and 1, the sample quantile with threshold q is used, whereas q=0.5 again results in the median as q= NA. |
| --- | --- |
| maxits | maximal number of iterations that should be executed. |
| convits | the algorithm terminates if the examplars have not changed for convits iterations. |
| lam | damping factor; should be a value in the range [0.5, 1); higher values correspond to heavy damping which may be needed if oscillations occur. |
| nonoise | If FALSE, adds a small amount of noise to prevent degenerative cases; if TRUE, this is disabled. |

###### Advanced parameters for "ISORANK"

ISORANK of NFPscanner relies on an Linux Executable of isorank-N program. The program isorank-n (based on based on isorank and multiway_kpartite) can find a global alignment of input networks. Given two networks with N1, N2 nodes each, it returns min (N1,N2) matches, each match corresponding to best-matching nodes from the two networks. Refer to [Citation](http://biotech.bmi.ac.cn/nfs/networksimilarityHelp#citationbodyA) for more details.

| K | max number of iterations. |
| --- | --- |
| thresh | the threshold for L1 norm of the change in the principal eigenvector between iterations. |
| alpha | the parameter that controls the relative weight of network and sequence data. 0 means only sequence data, 1 means only network data. The sweet-spot is usually around 0.6-0.8. |
| beta | the parameter that controls the relative weight of the first network to the second network. A beta of 0.5 weights both sets of networks equally and a beta of 0.75 weights the first network 3 times more than the second network. |
| maxveclen | since the eigenvalue computation is being performed on a sparse matrix and the eigenvector is also sparse, you can choose how many non-zero entries you want. In each iteration, the algorithm will keep only <maxveclen> of the largest values. |

###### Advanced parameters for "SPINAL"

SPINAL of NFPscanner relies on Linux Executable SPINAL program. Given two intereaction networks, the global network alignment algorithm "SPINAL" returns a one-to-one global mapping between their nodes. Refer to [Citation](http://biotech.bmi.ac.cn/nfs/networksimilarityHelp#citationbodyA) for more details about SPINAL.

| alpha | the parameter that controls the relative weight of network topology and sequence. 0 means only sequence data, 1 means only network data. |
| --- | --- |

###### Advanced parameters for "GHOST"

GHOST of NFPscanner relies on Linux Executable GHOST program. GHOST is a program that aligns two networks based on an algorithm detailed in "Global Network Alignment Using Multiscale Spectral Signatures" by Rob Patro and Carl Kingsford. Refer to [Citation](http://biotech.bmi.ac.cn/nfs/networksimilarityHelp#citationbodyA) for more details about GHOST.

| hops | The positive integer value for the radius of the subgraphs. The default value is 4. |
| --- | --- |
| nneighbors | The positive integer value for the number of nearest neighbors in H that each node in G will have a distance computed to. The default value is "all" which is coded as -1. |
| searchiter | The positive integer value for the number of local search iterations that should be performed after the initial alignment is complete. The process will terminate early if the last loop yielded no changes. The default value is 10. |
| ratio | The positive decimal value for the ratio of "bad-moves" allowed during the local-search phase of the alignment algorithm. The default value is 8.0. |

#### Choose a reference set of basic networks

Retrieved from seven pathway databases (KEGG, Reactome, NCI, Spike, PANTHER, Biocarta, HumanCyc), NFPscanner contains a total of 766 basic networks which are manually curated and revised by domain experts (arbitrarily, raw networks with less than 10 edges are excluded), and 49 biomedical relevant categories of reference sets extracted from these basic networks. The categories help users do network analysis with special research interest, such as regulatory circuits, signaling pathways, hormone regulation, disease or development, etc. On the NFPscanner analysis page, click to select one reference set from library. Otherwise click "learn more" to explore the library of reference sets on the resource page.


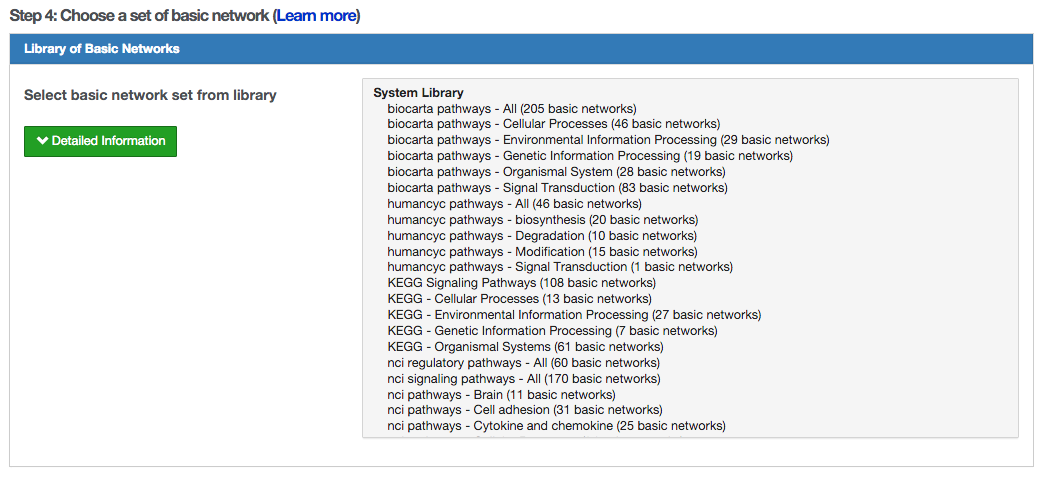


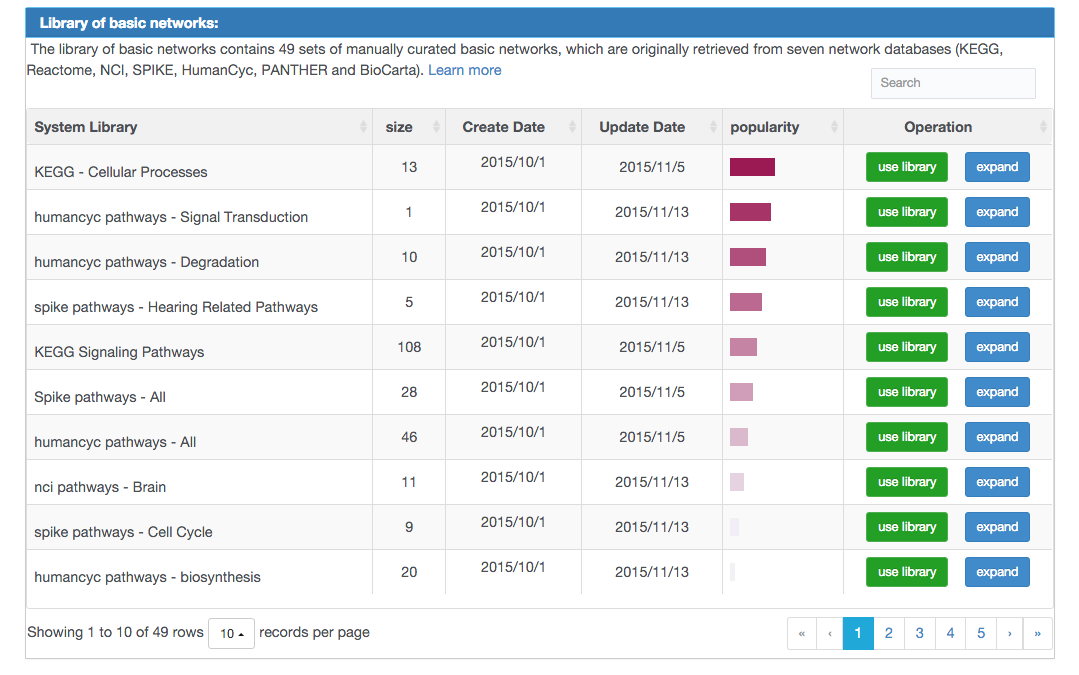


#### Submit a job to NFPscanner server

The last step is submitting the job to NFPscanner server. Click "Run Network Fingerprint Scan" button to submit. Optionally, user can enter an email address before submitting the job, notification emails with a web link to NFPscanner result will be sent from NFPscanner server to users. The analysis results will be saved on NFPscanner server.


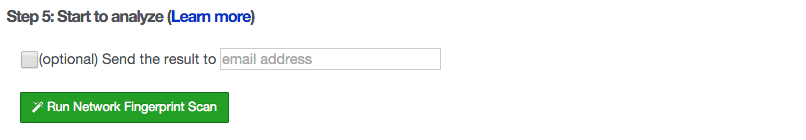


#### Monitor the status of a job

After submission is complete, you are redirected to the result page of NFPscanner. The page shows a summary of this NFPscanner program. Because a job can last hours or even days, NFPscanner summary includes a web link to the result page. You can bookmark the web page in browser. You can open the result page through the link to monitor the job status (waiting, queued, running, or done).


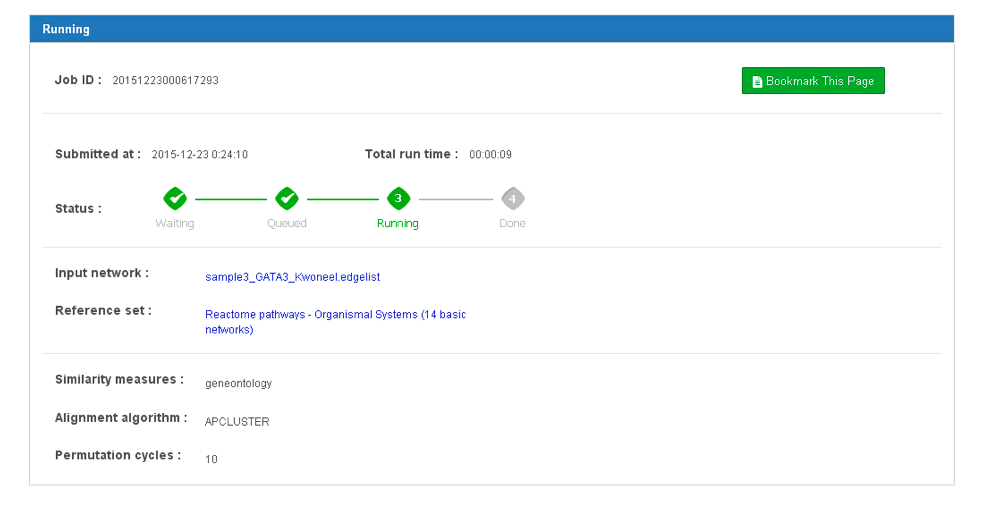


#### Understand Network Fingerprint result

After the network fingerprint computation is done,
1) If users provide their email when submitting the job, NFPscanner will send a notification email saying that the computation is completed. Click the web link in notification email to open result page.
2) If users haven’t close the webpage, network fingerprint result should appears automatically below the summary section. If the result page doesn’t refresh automatically, please refresh web page manually.
3) If users already bookmarked result page, click this bookmark to open result page again.
The NFPscanner visualizes network fingerprints on result page. This network fingerprint is made up of two parts:
The left part of result is "Fingerprint graph" of the network fingerprints. The fingerprints of multiple input network are visualized in different colors. Vertical axis indicates the similarity score between input networks and basic networks. Horizontal axis indicates the basic networks in order of categories. If you are scanning fingerprints for multiple input networks, the curves may overlap in certain regions of fingerprint plot. To see one of several fingerprint more clearly, use curve legend above fingerprint curves to turn off other curves and turn on the one curve. In this way, users can examine network fingerprints one by one. Mouseover the data points to see the corresponding similarity scores between one input network and one basic network. Click on the data points will expand the pairwise alignment details right below the fingerprint graph on the same page.
The right part of result is "Fingerprint data" table about these network fingerprints. The fingerprints in "Fingerprint graph" are linked with items in "Fingerprint data". When user mouse-over the fingerprint curves, the corresponding rows in "Fingerprint data" table are filtered and highlighted. The result table can be sort by basic network names and scores. You can also search a basic network name/keyword to find the scores of any specific network pairs. Mouseover a row will highlight the corresponding data point of fingerprint graph in the left part of result and simulanously show detailed information of the data point on the graph. Furthermore, the fingerprint graph also serves as an entrance for a closer investigation at network alignment level. Each data point on fingerprint curves represents a pairwise similarity score between one input network and one basic network. When multiple fingerprints differ in the range of certain basic networks, it suggests that these input networks have different functional associations with those biological pathways. Once some basic network or pathway is selected to make a further study, users can click the corresponding data point to perform pairwise alignment analysis between the input and basic networks.


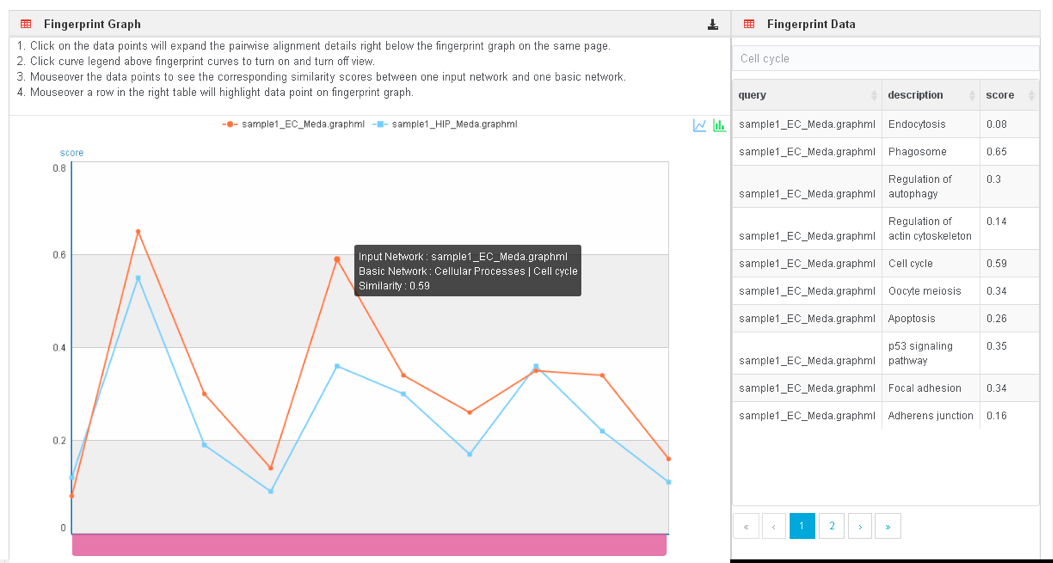


#### Explore pairwise alignment details

From the above network fingerprint results, you can identify interested basic network that you want to compare to input network. After click the corresponding data point, the two network alignment show up below the original fingerprint section. The result is made up of two parts:
In the "Alignment view", left side is the input network and right side is the basic network. Click network nodes to get annotation about the gene, which are hyperlinked to external databases, including ncbi gene, genecards, uniprot, genenames, ensemble, refseq.
In the "Alignment & Enrichment Data", there are three tabs: 1) "Network Alignment", which consists of similarity scores of gene clusters; 2) "Pathways", which are the enriched KEGG pathways in nodes of this input and basic network; 3) "Gene Ontology", which is the enriched Gene Ontology terms in nodes of two networks. The rows can be sorted and searched by keywords to find desired clusters, pathways and GO terms. Mouseover the rows and the corresponding nodes are highlighted in the "Alignment view".
If users want to see other pairwise network alignments, click on another data point on the fingerprint chart to update "Alignment view" and "Alignment & Enrichment Data".


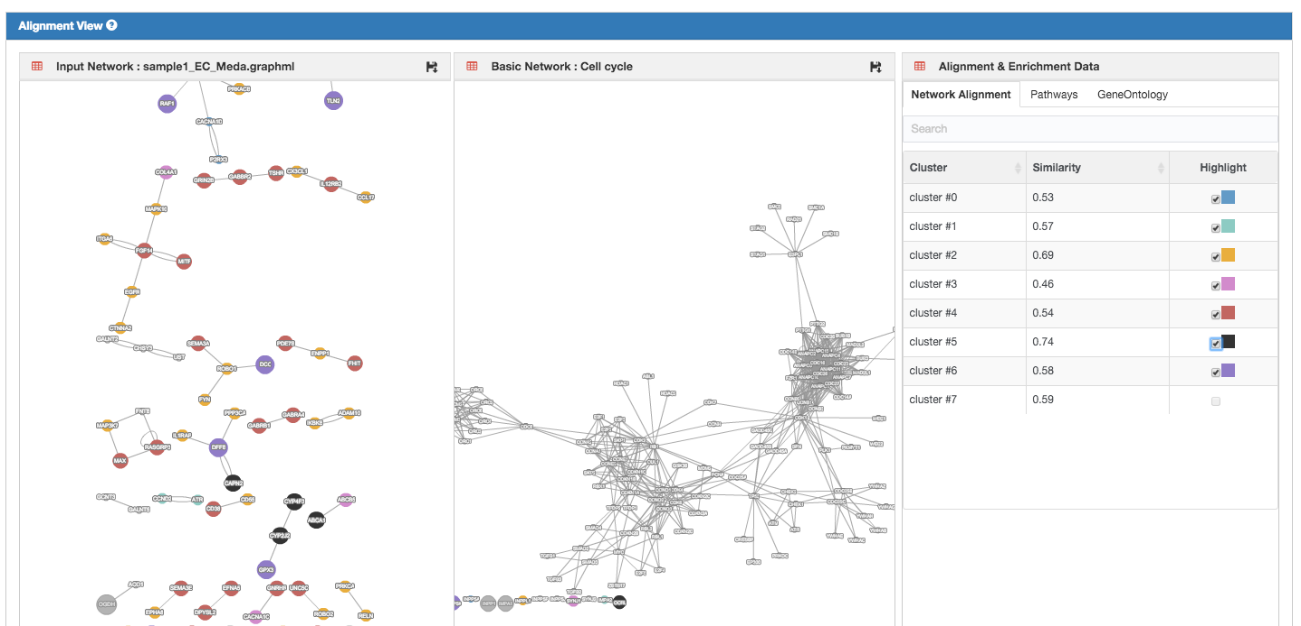


#### Download graphs and PDF report

The data chart, network graphs and analysis report can be exported to print, archive and share with others. Go to the top of the result page, click on "Download Report" button to export a complete PDF report of job information, parameter settings, reference set, and the analysis result including graphs and data table. In addition, there is a download button on upper-right corner in every chart and graph of the result page.


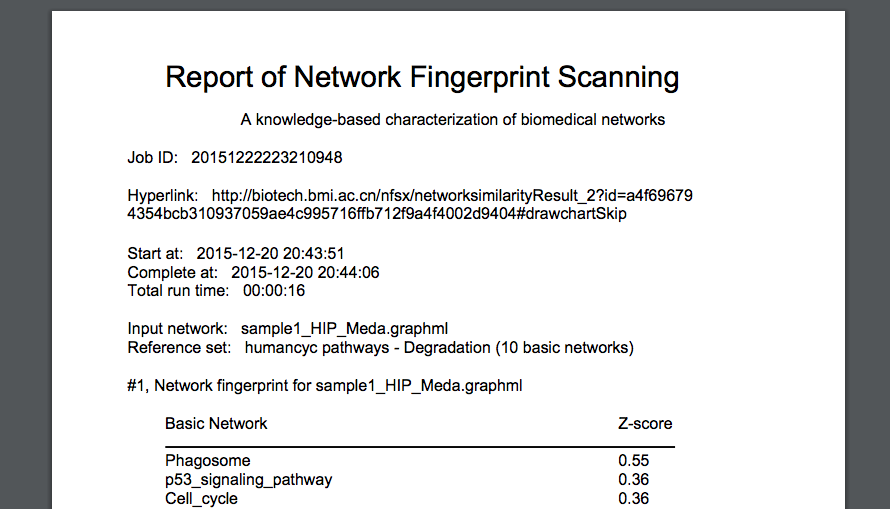


#### Disclaimer

NFPscanner is provided as a free public resource. We strive to reach a high level of quality, but can’t legally guarantee correctness, because all data is based on other public resources. The providers of this service do not guarantee the accuracy of any data networks or the original data on which that they are based. The providers of this service do not guarantee the suitability of databases or the services for any purpose.
Some of the original data used by the service may be subject to patent, copyright or other intellectual property rights that place restrictions on the use or redistribution of these data. It is the responsibility of the users of this service to ensure that their use of the service does not infringe on such rights and restrictions.
The providers of this service will attempt to maintain continuity of services and provide sufficient warning for changes or cancellations of services. However, the providers of this service do not accept responsibility for temporary or permanent unavailability of services.

#### Copyright

NFPscanner is licensed under a [Creative Commons Attribution-NonCommercial 4.0 International License](http://creativecommons.org/licenses/by-nc/4.0/). This means that you are free to copy, distribute, display and make non-commercial use of all results aquired from the NFPscanner web server, provided that appropriate credit is given. NFPscanner is intended to be a service to the community. It may be used free of charge in scientific and non-commercial software alike.

#### Acknowledgement

The web server acknowledges several open-source projects and codes: KEGGgraph, igraph, ggplot, clusterProfiler, graphite, cytoscape web, Bootstrap, jsTree, d3.js, e-charts, jsPDF. Special thanks to the authors of network analysis algorithms IsoRank-N2, SPINAL, GHOST, and APCluster, and authors of the example networks for their outstanding work on the research area.

#### Citation

If you will find the results produced by NFPscanner useful, please cite:

*Cui X, He H, He F, Wang S, Li F, Bo X. Network fingerprint: a knowledge-based characterization of biomedical networks. Scientific Reports. 2015 Aug 26;5:13286. doi: 10.1038/srep13286. PubMed PMID: 26307246; PubMed Central PMCID: PMC4549786*

If you are interested in alignment algorithms used by NFPscanner, please refer to:

**IsoRankN**

*Liao CS, Lu K, Baym M, Singh R, Berger B. IsoRankN: spectral methods for global alignment of multiple protein networks. Bioinformatics. 2009 Jun 15;25(12):i253-8. doi: 10.1093/bioinformatics/btp203. PubMed PMID: 19477996; PubMed Central PMCID: PMC2687957.*

**GHOST**

*Patro R, Kingsford C. Global network alignment using multiscale spectral signatures. Bioinformatics. 2012 Dec 1;28(23):3105-14. doi:10.1093/bioinformatics/bts592. Epub 2012 Oct 9. PubMed PMID: 23047556; PubMed Central PMCID: PMC3509496.*

**SPINAL**

*Aladag AE, Erten C. SPINAL: scalable protein interaction network alignment. Bioinformatics. 2013 Apr 1;29(7):917-24. doi: 10.1093/bioinformatics/btt071. Epub 2013 Feb 14. PubMed PMID: 23413436.*

**APCLUSTER**

*Bodenhofer U, Kothmeier A, Hochreiter S. APCluster: an R package for affinity propagation clustering. Bioinformatics. 2011 Sep 1;27(17):2463-4. doi:10.1093/bioinformatics/btr406. Epub 2011 Jul 6. PubMed PMID: 21737437.*

If you are interested in the example networks provided by NFPscanner, please refer to:

*Smith CL, Dickinson P, Forster T, Craigon M, Ross A, Khondoker MR, France R, Ivens A, Lynn DJ, Orme J, Jackson A, Lacaze P, Flanagan KL, Stenson BJ, Ghazal P. Identification of a human neonatal immune-metabolic network associated with bacterial infection. Nat Commun. 2014 Aug 14;5:4649. doi: 10.1038/ncomms5649. PubMed PMID: 25120092; PubMed Central PMCID: PMC4143936.*

*Kim K, Yang W, Lee KS, Bang H, Jang K, Kim SC, Yang JO, Park S, Park K, Choi JK. Global transcription network incorporating distal regulator binding reveals selective cooperation of cancer drivers and risk genes. Nucleic Acids Res. 2015 Jul 13;43(12):5716-29. doi: 10.1093/nar/gkv532. Epub 2015 May 22. PubMed PMID: 26001967; PubMed Central PMCID: PMC4499150.*

*Meda SA, Koran ME, Pryweller JR, Vega JN, Thornton-Wells TA; Alzheimer's Disease Neuroimaging Initiative. Genetic interactions associated with 12-month atrophy in hippocampus and entorhinal cortex in Alzheimer's Disease Neuroimaging Initiative. Neurobiol Aging. 2013 May;34(5):1518.e9-18. doi: 10.1016/j.neurobiolaging.2012.09.020. Epub 2012 Oct 27. PubMed PMID: 23107432; PubMed Central PMCID: PMC3570748.*

*Han S, Yang BZ, Kranzler HR, Liu X, Zhao H, Farrer LA, Boerwinkle E, Potash JB, Gelernter J. Integrating GWASs and human protein interaction networks identifies a gene subnetwork underlying alcohol dependence. Am J Hum Genet. 2013 Dec 5;93(6):1027-34. doi: 10.1016/j.ajhg.2013.10.021. Epub 2013 Nov 21. PubMed PMID: 24268660; PubMed Central PMCID: PMC3853414.*

#### FAQ

What is edge list format?

The edgelist format is a simple text file, with one edge in a line, the two vertex ids separated by a space character.


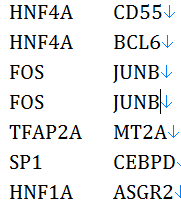


What is igraph?

The igraph is a collection of network analysis tools. It can also import and export many network formats. In the help section [Make an input network from other formats"](http://biotech.bmi.ac.cn/nfs/networksimilarityHelp#igraphclassbodyA), we describes how to export edgelist and graphml from R. Visit http://igraph.org/ for more about igraph.

What is network fingerprint?

In the [Cui et.al 2015](http://biotech.bmi.ac.cn/nfs/networksimilarityHelp#citationbodyA), a biomedical network is characterized as a spectrum-like vector called "network fingerprint", which contains similarities to basic networks. This knowledge-based multidimensional characterization provides a more intuitive way to decipher molecular networks, especially for large-scale network comparisons and clustering analyses.

Which alignment algorithms should I use in NFPscanner analysis?

NFPscanner current implemented four alignment algorithms. We have no discrimination on the performance of these algorithms in terms of network fingerprint analysis and network alignment. Among them, Affinity Propagation clustering is used in original publication of network fingerprint. Refer to [Citation](http://biotech.bmi.ac.cn/nfs/networksimilarityHelp#citationbodyA) for more information about alignment algorithms.

What basic networks should I select from library in NFPscanner analysis?

NFPscanner is knowledge-based computational framework to decipher biomedical networks by making systematic comparisons to reference sets of well-studied "basic networks". The fingerprints of biomedical networks is associated with users’ choice of reference sets. We recommend users to browse "Resource" page and find the most appropriate reference set relating to their research topics.
