# Supplementary figures and images for: NFPscanner: a webtool for knowledge-based deciphering of biomedical networks
Source: BMC Bioinformatics. 2017 May 18;18:262. doi: 10.1186/s12859-017-1673-1 (PMC5437514; doi:10.1186/s12859-017-1673-1)

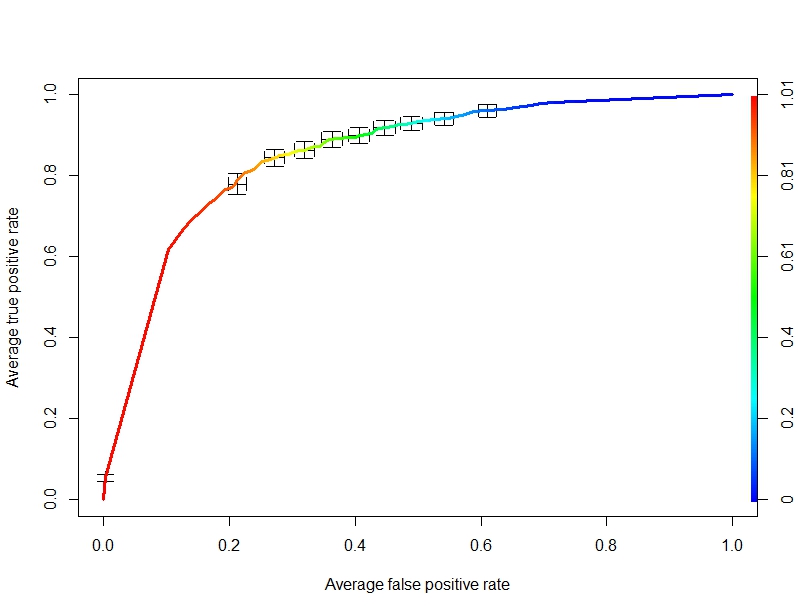

Supplement: Supplementary file 3 — Performance of algorithm and parameter combinations on the same input data set. Table S2. AUC value of networks fingerprint results for 73 KEGG diseases datasets. Figure S1. Average ROC curves derived from Table S2. Table S3. Fingerprints data file in Case Studies. (ZIP 95 kb) [file 12859_2017_1673_MOESM3_ESM.zip › Supplemental File 3/Fig S1. Average ROC curve_v1.jpeg]
